# Veering to a Continuous Platform of Fused Deposition Modeling Based 3D Printing for Pharmaceutical Dosage Forms: Understanding the Effect of Layer Orientation on Formulation Performance

**DOI:** 10.3390/pharmaceutics15051324

**Published:** 2023-04-23

**Authors:** Vineet R. Kulkarni, Jaidev Chakka, Faez Alkadi, Mohammed Maniruzzaman

**Affiliations:** Pharmaceutical Engineering and 3D Printing (PharmE3D) Lab, Division of Molecular Pharmaceutics and Drug Delivery, College of Pharmacy, The University of Texas at Austin, Austin, TX 78705, USA; vineetkulkarni@utexas.edu (V.R.K.); jc@austin.utexas.edu (J.C.); faez@austin.utexas.edu (F.A.)

**Keywords:** 3D printing, additive manufacturing, hot-melt extrusion, amorphous solid dispersion, continuous printing, layer orientation, fused deposition modeling

## Abstract

Three-dimensional (3D) printing of pharmaceuticals has been centered around the idea of personalized patient-based ‘on-demand’ medication. Fused deposition modeling (FDM)-based 3D printing processes provide the capability to create complex geometrical dosage forms. However, the current FDM-based processes are associated with printing lag time and manual interventions. The current study tried to resolve this issue by utilizing the dynamic *z*-axis to continuously print drug-loaded printlets. Fenofibrate (FNB) was formulated with hydroxypropyl methylcellulose (HPMC AS LG) into an amorphous solid dispersion using the hot-melt extrusion (HME) process. Thermal and solid-state analyses were used to confirm the amorphous state of the drug in both polymeric filaments and printlets. Printlets with a 25, 50, and 75% infill density were printed using the two printing systems, i.e., continuous, and conventional batch FDM printing methods. Differences between the two methods were observed in the breaking force required to break the printlets, and these differences reduced as the infill density went up. The effect on in vitro release was significant at lower infill densities but reduced at higher infill densities. The results obtained from this study can be used to understand the formulation and process control strategies when switching from conventional FDM to the continuous printing of 3D-printed dosage forms.

## 1. Introduction

The 3D printing of personalized medication and pharmaceutical dosage forms is a promising field of research that has attracted the attention of multiple research groups [1]. Its potential has been exploited by the pharmaceutical industry, which has led to an exponential rise in the number of research publications and patents [2,3]. The approval of Apprecia’s 3D-printed dosage form, Spritam™, by the United States Food and Drug Administration (FDA) has further increased the interest in the application of different 3D printing techniques to formulate dosage forms [4]. This idea has been centered around the idea of a personalized patient-based and on-demand supply of medications [1]. Three-dimensional printing or additive manufacturing can be defined as a process of fabricating 3D objects from a digital file in a layer-by-layer fashion [5]. Even though the process of 3D printing can be defined by its final product, multiple technologies work on different principles to manufacture the 3D objects [6,7,8]. Manufacturing 3D-printed objects using a polymeric filament is one such approach [4].

Fused deposition modeling (FDM), a material extrusion-based 3D printing, involves the additive deposition of polymeric filaments extruded through a computer-controlled deposition nozzle at high temperatures, providing the capability to create complex geometries as well as 3D models with controlled composition and architecture [9,10,11]. FDM has been the most evaluated 3D printing technique for the manufacturing of pharmaceutical dosage forms [12]. The FDM technique predominantly utilizes thermoplastic polymers as a feedstock that has paved the way for processing pharmaceutical compositions [13,14]. Owing to the simplicity of the equipment coupled with the diverse choice of excipients and ease of operation, producing dosage forms with complex geometries for personalized therapy is a possibility [12].

The current FDM-based processes used in pharmaceutical dosage form development involve a pre-print lag time for heating the nozzles, followed by a post-print lag time for cooling the nozzle every time before starting the next print [15]. This lag time also involves a manual intervention to remove the printed dosage forms and then start a second print. This practical limitation is majorly due to the confined print bed area of most FDM 3D printers, which also limits the applicability to a personalized on-demand application [15,16,17]. Furthermore, the involvement of a manual intervention followed by post-processing steps and collection makes it unfavorable for scaling up and bringing the printed dosage forms to the market [18,19].

**Figure 1:**
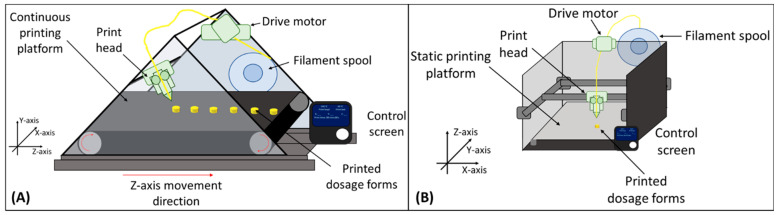
(**A**) Continuous platform for fused deposition modeling (FDM)-based 3D printing and (**B**) conventional batch process for FDM-based 3D printing.

The dynamic *z*-axis continuous 3D printing mechanism has a large surface conveyor belt that moves forward as the print progresses. The extrusion nozzle of the printhead for the continuous 3D printing process is oriented at an angle <90 degrees to the build surface compared to the vertical orientation (90 degrees) in the current FDM printers. The adaptation of this continuous 3D printing system would bypass the pre-print and post-print lag times, and thereby reduce the manual intervention every time. The conveyor belt moves along the *z*-axis as the print progresses at an angle (25–60 degrees between the extruder and build surface (Figure 1A)), which helps to minimize and eliminate the use of support structures during printing [20,21]. This paper aims to set up and evaluate the continuous platform to understand the effect of the printed layer orientation on drug release and tablet mechanical strength, the two important parameters for drug dosage form performance, as compared to the conventional FDM printers that are limited to the batch printing process. 

## 2. Materials and Methods

### 2.1. Materials

The drug, fenofibrate (FNB) (Tokyo Chemical Industry Co., Ltd., Tokyo, Japan, Lot no: WQYKC-PW), and AquaSolveTM HPMC-AS LG (Ashland Specialty Ingredients, Wilmington, NC, USA, Lot no. 55G-910001), were used in this study as an active drug and as polymeric carriers, respectively. Sodium phosphate monobasic, sodium hydroxide, and sodium chloride were purchased from Fisher Scientific (Hampton, NH, USA) for buffer preparation. The 3D printers were purchased and modified in-house. All other chemicals, solvents, and reagents used in this study were of analytical or high-performance liquid chromatography (HPLC) grade and obtained from Fisher Scientific (Waltham, MA, USA).

### 2.2. Solid-State Analysis (Pre-Printing Process)

#### 2.2.1. Thermogravimetric Analysis (TGA)

A TGA/DSC 1 (Mettler-Toledo, Schwerzenbach, Switzerland) piece of equipment was used to understand the thermal properties of the pure crystalline drug, polymers, and drug–polymer physical mixtures (PM). The samples were loaded into an open crucible, placed in the furnace, and heated from 35 to 350 °C at a rate of 10 °C/min. The TGA was run under an ultra-purified nitrogen environment at a 50 mL/min purge gas flow rate. The data was collected and analyzed using the STAR software (Mettler-Toledo, Switzerland). 

#### 2.2.2. Differential Scanning Calorimetry (DSC) Analysis

A Q20 DSC unit (TA^®^ Instruments, New Castle, DE, USA) was used to conduct modulated differential scanning calorimetry for the pure crystalline drug, polymers, PM, and the manufactured drug-loaded extrudates (EXT). The thermal trends seen from the raw material plots were used to set the processing parameters for the HME process to manufacture the EXT of interest. Briefly, 5–10 mg of the samples were weighed in T-zero aluminum pans (DSC Consumables Incorporated, Austin, MN, USA) using a calibrated microbalance. The samples were heated from 35 to 180 °C at a 10 °C/min ramp rate and the temperature was modulated by 0.50 °C every 30 s, under a nitrogen flow of 50 mL/min. The collected data were analyzed and plotted as temperature (°C) versus heat flow (W/g). 

#### 2.2.3. Powder X-Ray Diffraction (XRD) Analysis 

The FNB crystallinity in the formulated extrudates was evaluated using a benchtop Rigaku MiniFlex 600 (Rigaku Corporation, Tokyo, Japan). The instrument (equipped with a Cu Kα radiation source) was set to 15 mA with a 40 kV voltage. The samples (pure crystalline drug, polymer, PM, and the drug-loaded EXT crushed into a fine powder) were evenly spread into the holder and analyzed over a 2θ range of 5–60° with a scan speed of 2°/min and a step size of 0.02°/min.

#### 2.2.4. Polarized Light Microscopy (PLM)

The FNB residual crystalline in the EXT samples was analyzed along with the raw materials using an Olympus BX53 polarized photomicroscope (Olympus America Inc., Webster, TX, USA). The microscope was equipped with a Bertrand lens. The birefringence in crystalline substances was observed under 10× magnification. The images captured using a QICAM Fast 1394 digital camera (QImaging, Surrey, BC, Canada) with a 530-nm compensator (U-TP530, Olympus^®^ Corporation, Shinjuku City, Tokyo, Japan) were analyzed using Linksys 32 software^®^ (Linkam Scientific Instruments Ltd., Tadworth, UK).

#### 2.2.5. Fourier Transform Infrared Spectroscopy (FTIR)

The intermolecular interactions between FNB and the carrier matrix were investigated using an FTIR analysis (iS50 FTIR equipped with a SMART OMNI-Sampler, ThermoFisher Scientific, Waltham, MA, USA). The FNB, HPMC AS LG, PM, and crushed EXT were analyzed from 4000–700 cm^−1^, at a resolution of 4 cm^−1^ for % transmittance with 64 scans per run. The background was collected before every run. Weak intermolecular interactions were analyzed and assessed on the OMNIC^TM^ series software (ThermoFisher Scientific, Waltham, MA, USA) for the collected spectra.

### 2.3. Hot-Melt Extrusion (HME) 

**Figure 2:**
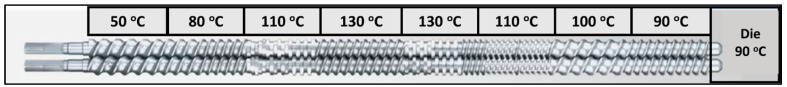
Temperature profile setup for hot-melt extrusion (HME) of filaments for 3D printing.

The preliminary screening of polymers for FNB was conducted using Hansen solubility parameters and thermal analysis of API and excipients. Based on the thermal investigation, the processing parameters shown in Figure 2 were selected. A 20% *w*/*w* of FNB was mixed with 80% *w*/*w* HPMC AS LG using geometric dilution. Filaments for 3D printing were extruded using a Leistritz ZSE 12 HP-PH 12 mm twin screw co-rotating HME extruder (Leistritz Advanced Technologies Corp., Nuremberg, Germany). The optimized temperature profile and the screw design used for the extrusion process based on the thermal investigation are shown in Figure 2. The blend was introduced into the feeding section of the extruder using a calibrated volumetric feeder (Brabender twin screw feeder with stirring agitators, Brabender Technologie, Mississauga, ON, Canada) at a feeding rate of 3 g/min. The process was run at 50 rpm. A 1.75 mm die was used for the extrusion process to match the requirements of the FMD printer. The diameter of the filaments was monitored using a Vernier caliper, and the filaments collected post-extrusion were stored in a validated desiccator for further use and characterization. 

### 2.4. Designing and FDM 3D Printing of Formulations

The 3D designs for the print were designed using Solidworks (SolidWorks Corp., Dassault Systèmes, Vélizy-Villacoublay, France) where the model was designed in a cylindrical shape with an 8 mm diameter and 5 mm height. Three different infill densities, 25, 50, and 75%, were used for the study. The designs were sliced using a manufacturer slicing software (Stratasys, Rehovot, Israel) for printing using the batch 3D printer and using Creality Cura (Shenzhen Creality 3D Technology Co, Ltd., Shenzhen, China) software for printing using the continuous 3D printer. All other design parameters were kept the same for printing using both systems. The layer thickness was set to 0.1 mm with a grid infill printing pattern. A 0.4 mm print head was used with the printing temperature set at 140 °C and the printing speed was 20 mm/s. The bed temperature was set to 60 °C. 

### 2.5. Assessment of Printed Tablets

A digital caliper (Neiko 01407A, VWR, Radnor, PA, USA) was used to measure the accuracy and reproducibility of the dimensions of the printed dosage forms using the two printing systems. A Mettler-Toledo ME-TE (Mettler-Toledo, LLC., Columbus, OH, USA) analytical balance was used to obtain the weight of the printed tablets. A Dino-Lite optical microscope (AnMo Electronics Corporation, Torrance, CA, USA) was used to capture images of the dosage forms. 

### 2.6. Evaluation of Dosage Form Breaking Force

The strength of the printed tablets or printlets was evaluated in two different directions. The printlets in two batches, namely 45° print layer (printed in a continuous process) and 0° (printed in a batch process), were subjected to a breaking force using a manual tablet hardness testing instrument (PTB-M, Pharma Test, Hainburg, Germany) along their horizontal and vertical axes to understand the effect of the print layer orientation on the printed final dosage form. The force needed to break the print or cause its collapse was recorded and compared against other groups. The study was conducted using 10 printlets per group. Both groups for every infill density were compared against each other to compare the trend with increasing infill densities. The images during the setup and evaluation of the breaking force were captured by using digital microscopy (Dino Light, Torrance, CA, USA). 

### 2.7. In Vitro Release Performance Testing 

The in vitro performance testing was carried out using a US Pharmacopeia (USP) type II dissolution apparatus (Vankel VK 700, Agilent Technologies, Santa Clara, CA, USA). A total of 500 mL of phosphate buffer (0.1 M, pH 6.8) was added to the dissolution vessels. The media was maintained at 37 ± 0.5 °C and stirred at 75 rpm. An autosampler (Vankel VK 8000, Agilent Technologies, USA) was used to withdraw 1 mL of media at predetermined time points, which was then replaced with a fresh phosphate buffer. These samples were filtered (10 µm polyethylene dissolution filters, Fisher Scientific, Hampton, NH, USA). The collected samples were diluted two-fold with acetonitrile (HPLC grade, Sigma-Aldrich, St. Louis, MO, USA) and the drug amount was estimated using the described method of analysis. The study was carried out in triplicates (n = 3) for all batches.

The drug release profiles of the samples were compared using a model-independent difference factor (*f*_1_) and similarity factor (*f*_2_), where *f*_1_ calculates the percent (%) difference between two curves at each time point and is a measurement of relative error between the two curves, and *f*_2_ measures the comparison of the percent (%) dissolution among two curves and is the “Log reciprocal square root conversion of the sum-of-squared-error” [22].

The difference factor (*f*_1_) was calculated using the following equation:

(1) $$f_{1}=\{[\sum_{t=1}^{n}\left|R_{t}-T_{t}\right|]/[\sum_{t=1}^{n}R_{t}]\}\cdot 100$$

The similarity factor was calculated using the following equation:

(2) $$f_{2}=50\cdot log\{[1+\left(\frac{1}{n}\right)\sum_{t=1}^{n}{{\left(R_{t}-T_{t}\right)}^{2}]}^{-\frac{1}{2}}\cdot 100\}$$

where *n* is the number of time points, *R_t_* is the percent drug release of the reference sample (batch printing process) at time point *t*, and *T_t_* is the percent drug release of the test sample (continuous printing process) at time point *t*. A difference factor (*f*_1_) close to zero (≤15) indicates minimal differences between the curves, and a similarity factor (*f*_2_) close to 100 (≥50) indicates closeness between the values of the test and reference samples.

### 2.8. Analytical Method

The concentration of FNB in the samples’ post-release studies was analyzed using reverse-phase high-performance liquid chromatography (RP-HPLC) analysis (Thermo Fisher Vanquish HPLC system, Thermo Fisher, Waltham, MA, USA). A stainless-steel C-18 column (250 × 2.1 mm, 2 µm particle size) (Avantor ACE^®^ EXCEL 3 C-18-AR column, VWR International, Radnor, PA, USA) was used for the analysis. The mobile phase was prepared using deionized water with 0.1% ortho-phosphoric acid (pH 2.5) and acetonitrile (ACN) at a 70:30 ratio. The flow rate was set to 1 mL/min and the injection volume was set at 10 µL. The run time for each sample was 10 min at 30 °C. The retention time for the drug was observed at 7.450 min. The detector used in this analysis was anultraviolet–visible (UV–Vis) spectrophotometer (Vanquish HPLC system, Thermo Fisher, Waltham, MA, USA) at 290 nm. The estimation of the collected data was performed using a calibration curve ranging from 1 to 128 µg/mL (R^2^ = 0.999). 

### 2.9. Statistics

Analysis of variance (ANOVA) followed by Tukey’s multiple comparison tests were conducted to compare and check if there was any statistical difference between the groups in terms of the breaking force required, with the *p*-value set to <0.05 for significance. 

## 3. Results

### 3.1. Pre-Formulation Characterization and Thermal Assessment 

**Figure 3:**
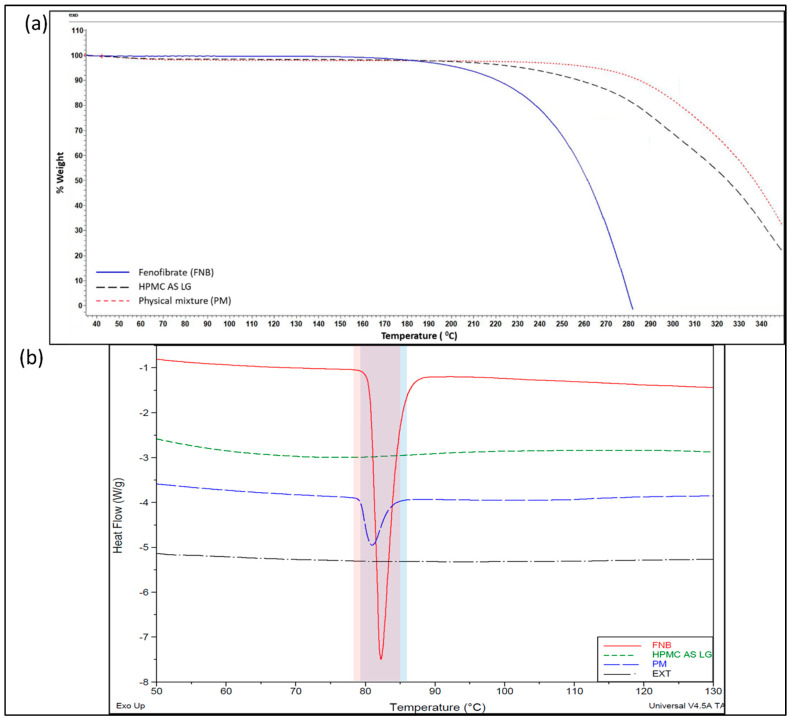
(**a**) Thermogravimetric analysis (TGA) curves for the pure drug, fenofibrate (FNB) (solid blue line), HPMC AS LG (dashed black line), and physical mixture (PM) (dotted red line); (**b**) DSC curves for fenofibrate (FNB), HPMC AS LG, physical mixture (PM), and extruded filaments (EXT), regions highlighted under blue and red bars indicate melting region for pure FNB and FNB in PM respectively; purple (the common region between both bars) displays a slight depression in the melting point for FNB.

HME and FDM 3D printing are associated with high processing temperatures [23]. Understanding the degradation profiles of the components involved in the study is important to set optimal processing conditions to prevent any degradation. From the blue line of the TGA plot, as shown in Figure 3a, FNB exhibited a weight loss starting around 180 °C when heated from 35 to 350 °C. Additionally, the physical mixture (PM) line shows better stability with degradation starting around 240 °C. 

Thermal runs using the DSC as seen in Figure 3b showed that pure FNB melts at 79 and 78 °C in its physical mixture of interest, respectively, for the extrusion process. The regions highlighted under the blue and red bars indicate the melting regions for pure FNB and FNB in PM, respectively, and purple (the common region between both bars) displays a slight depression in the melting point for FNB. This does not appear to be significant given the closeness of values as mentioned above for the broad processing temperature profiles, given that the glass transition temperature of the polymeric matrix, HPMC AS LG, was observed between 120 and 130 °C. These thermal events help us process the physical mixture using the HME as per the screw designs and thermal profiles that are shown in Figure 2 for making FNB-loaded filaments for 3D printing. 

In addition, the compatibility of drug–polymer miscibility was evaluated by applying the theoretical structural orientation-based prediction model of Hansen Solubility Parameters (Δδ = 3.40 MPa^1/2^ i.e., <7) for fenofibrate (δt = 20 MPa^1/2^) and HPMC AS polymer (δt = 24 MPa^1/2^) (where δ(MPa^1/2^) is the (total) solubility parameter) [14,24,25]. From the Δδ values, it was confirmed that FNB and HPMC AS LG pair were highly likely to be miscible and would form a solid dispersion. This theoretical evaluation of miscibility during the pre-formulation stages is important to predict the possibility of converting the crystalline drug to its amorphous state to form an amorphous solid dispersion (ASD) [24]. The balance needs to be achieved between the intramolecular interaction energy within a drug and the intermolecular drug–polymer interactions, where the polymer acts as the carrier matrix in this case [26]. 

### 3.2. Processing FNB-Loaded Drug Filaments Using the HME Process

Three-dimensional printable filaments with proper physical and chemical properties were successfully obtained via an optimized HME process based on the temperature profiles used as shown in Figure 2. The temperature ranges for the different zones was optimized to ensure that the final formulated filaments had an optimal balance between flexibility and brittleness to enable FDM-3D printing of the dosage form. Even though a 1.75 mm round-shaped die was used for the process, the collected filaments had a diameter of 1.65 ± 0.05 mm. This can be attributed to the thinning contributed by the forces applied by the puller, and to the immediate swelling of the filaments post-heating from the extrusion process followed by the contraction on cooling. The swell ratio increases as the processing temperature and the temperature of the extrudates at the outlet increase [27]. 

### 3.3. Characterization of FNB-Loaded Extruded Filaments

The extruded filaments were glassy in appearance and had a smooth surface. DSC studies were conducted to determine the amorphization of the FNB under the processing conditions used. The polymeric matrix, HPMC AS LG, used in this study acted as the dispersion medium to solubilize the drug when the polymer was in the molten state. As seen in Figure 3b, no endothermic peaks corresponding to the melting peak of FNB can be seen in the case of the extruded filaments (EXT), indicating that FNB might dissolve or be dispersed in the matrix before reaching its melting points. This indicates that the drug might have been converted to its amorphous state during the extrusion process. To support and confirm this, a further analysis was performed using PXRD and PLM [28]. 

**Figure 4:**
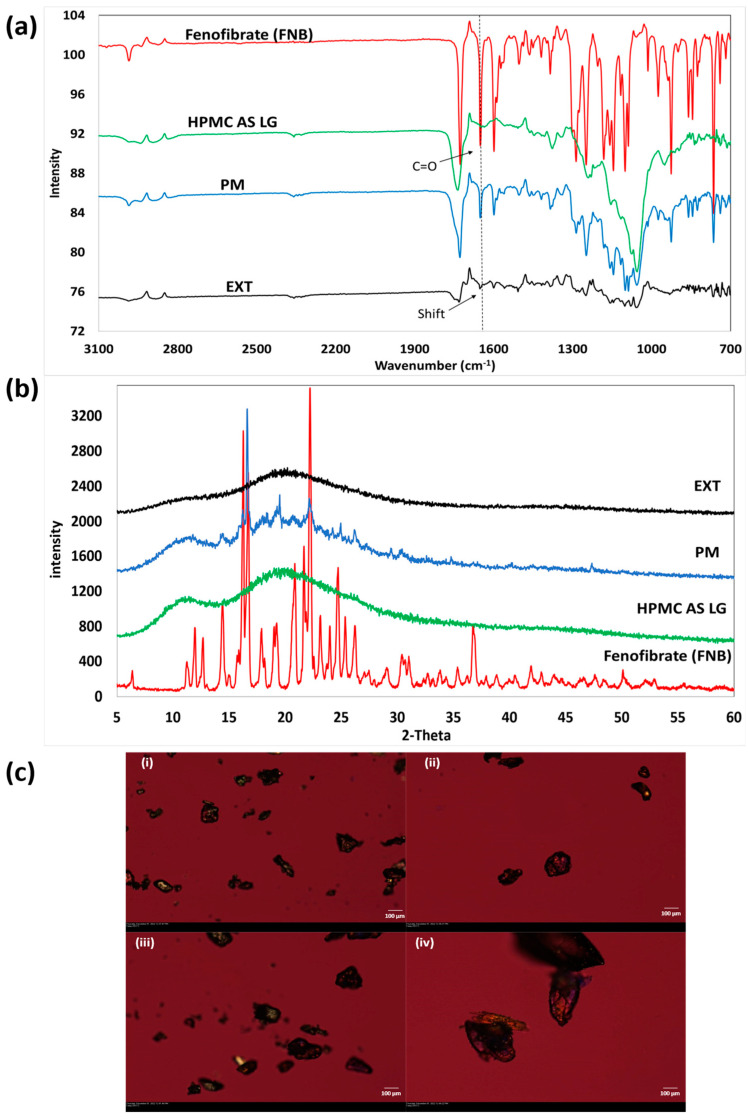
(**a**) Powder X-ray diffractograms of fenofibrate (FNB), HPMC AS LG, physical mixture (PM), and extruded filaments (EXT); (**b**) Fourier transform infrared spectroscopic (FTIR) spectra (PM: physical mixture, EXT: FNB-loaded extruded filaments); (**c**) polarized light microscopy (PLM) of: (**i**) pure crystalline fenofibrate (FNB), (**ii**) HPMC AS LG, (**iii**) 20% FNB + HPMC AS LG physical mixture (PM), and (**iv**) FNB-loaded extruded filaments (EXT), all taken under a light background using 530 nm compensator.

As seen in Figure 4, the crystalline characteristics of the pure drug, FNB, can be seen by the distinct peaks seen around a 2-theta of 16.26, 16.70, 22.2, 24.7, and 47.7° [29]. HPMC AS LG on the other hand showed no distinct peaks and exhibited the distinct halo corresponding to its amorphous nature. The PM showed the same distinct peaks that were exhibited by FNB but with reduced intensity indicating the crystalline nature of the drug in the PM before processing. Post-processing the EXT did not show the presence of peaks corresponding to the drug and exhibited a halo, supporting our claims of the amorphization of FNB in the polymeric matrix [30,31,32]. 

The FTIR analysis was conducted to understand the intramolecular interactions between FNB and HPMC AS LG in the extruded filaments after confirming the formation of ASD. As seen in Figure 4b, the peaks seen in the pure drug spectra seen around 1650 cm^−1^ correspond to ‘C=O’ stretching because of the ester group [33]. These peaks can be seen in the PM and extrudate spectra as well with reduced intensity. A slight shift towards the higher wavenumber can be seen in these peaks in the spectra for the extrudate, which may be due to an interaction with the polymer. This may be contributing to the stabilization of ASD [26]. 

The PLM analysis helped to observe the distribution of the drug in the polymeric matrix and any traces of crystallinity in the extruded filaments. As seen in Figure 4c, the bulk drug exhibits birefringence due to its crystalline nature, which gives it the property to refract light. Additionally, HPMC AS LG showed light polarization as well, which might have been because of its semi-crystalline structures. The birefringent pattern, which was seen in the PM as well, was absent in the EXT, indicating the absence of crystallinity in the processed filaments. This along with the observations from the DSC and XRD analysis helps confirm the amorphization of the FNB post-extrusion process and formation of ASDs. Any traces of birefringence observed in the ASDs under PLM are due to the semi-crystalline backbone of HPMC AS LG [34]. 

### 3.4. Print Quality Comparison between the Two Processes 

**Figure 5:**
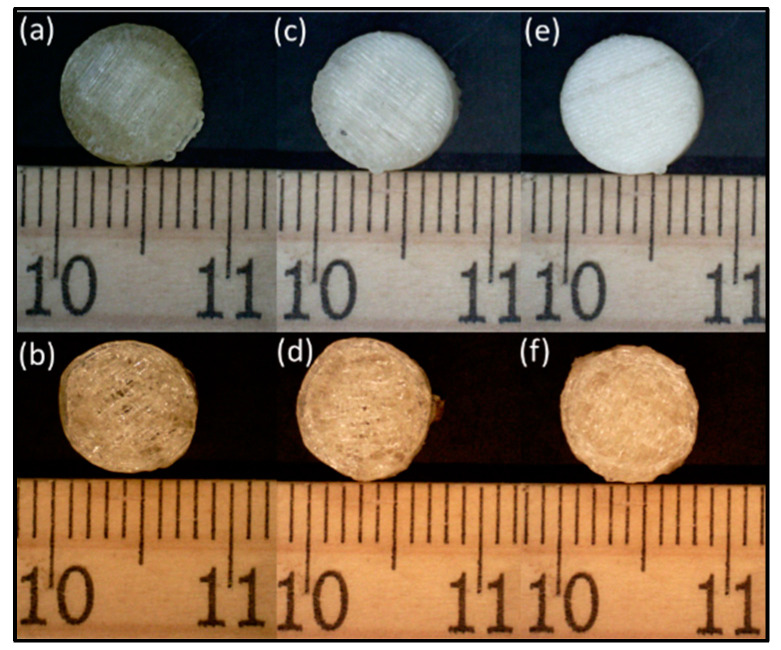
Digital microscopy images of printed tablets using continuous and batch printing: (**a**) 25% infill with a continuous process, (**b**) 50% infill with a continuous process, (**c**) 75% infill with a continuous process, (**d**) 25% infill with the batch process, (**e**) 50% infill with the batch process, (**f**) 75% infill with batch process.

The printlets obtained using the continuous printing method displayed a much smoother and more regular surface as compared to the prints obtained using the batch process as seen in Figure 5. In addition, the structural integrity and adherence to the indicated shape were higher in the case of the former prints (Table 1). 

The quality of the prints was much better for the printlets obtained using the continuous method when checked visually. When printing using a conventional batch process method at a 0° axis, the initially printed bottom layers tended to show an increase in the circumference and a decrease in the layer thickness due to the pressure exerted by the gravitational force from the layers printed on top of these initial base layers. This contributed to an irregular shape, which reduced the visual appeal of the printed printlets, which plays a key role in patient compliance with the medication regimens. This phenomenon was not seen in the case of our continuous printing system, where the printed parts moved forward, making space for the next layer to be printed along the 45° axis, and thus avoiding a direct exertion of pressure on the previously printed layers. 

### 3.5. Effect of Layer Orientation on Printlet Strength 

**Figure 6:**
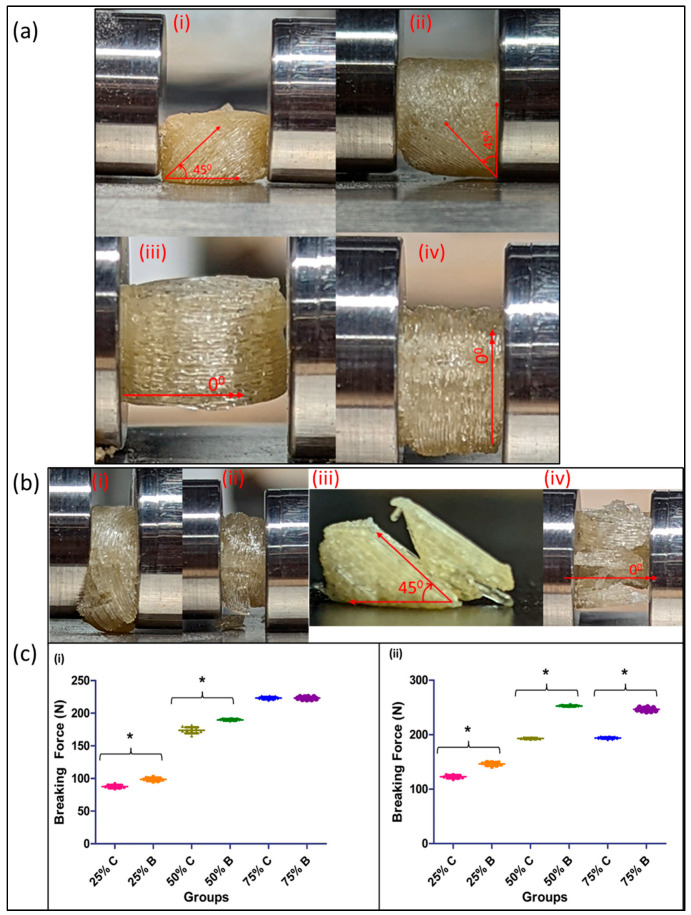
(**a**) Breaking force test comparison setup for prints fabricated using continuous process: (**i**) horizontal testing setup, (**ii**) vertical testing setup; and prints fabricated using batch process: (**iii**) horizontal testing setup, (**iv**) vertical testing setup; (**b**) collapse and breakage of tablets post-breaking force test of (**i**) prints fabricated using the continuous process in vertical orientation test, and (**ii**) prints fabricated using a batch process in vertical orientation test; and (**iii**) prints fabricated using a continuous process in horizontal orientation test, and (**iv**) prints fabricated using a batch process in horizontal orientation test; and (**c**) breaking force test comparison: (**i**) horizontally tested and (**ii**) vertically tested, for printlets printed using continuous printing process (C) and batch printing process (B) across 25% (25C and 25B), 50% (50C and 50B), and 75% (75C and 75B) infill densities. The data are represented by mean ± standard deviation for n = 10 samples. The significance of the difference is * *p* < 0.005.

The effect of the breaking force on the printlet varied based on the layer orientation and the direction in which they were assessed (Figure 6a). The results can be spoken about in two cases:

- Testing when the printlets are placed horizontally: At both a 25% and 50% infill density, the print differences proved to be statistically significant as compared to the 75% infill density prints, where the difference in the force required to break them was not significant. In addition, as the infill density increased, the force required to break the printed tablet increased, which correlated to an increased strength due to reduced void spaces in the prints (Figure 6c). The printlets formulated using the continuous method broke with the print splitting in two pieces and with the split occurring along the axis of printing (45°), across all infill densities tested for the study. The reason for this split along the axis can be attributed to the fact that, when a force is applied at a 45° angle, it becomes resolved into two components, one that acts in line and the other along the axis of the printed angle. This split in the applied force prevents the crushing of the tablet and in turn, ends up splitting it along its printed axis. Comparatively, when printed using the conventionally used pharmaceutical FDM printing process, the tablets when tested in the horizontal orientation were crushed into small fragments.
- Testing when the printlets are placed vertically: As compared to the horizontal orientation for testing, the force required to break the printlet was significantly different across all the infill densities tested in this study. The force required to break a printlet with layers printed at 45° axes is greater as compared to the force required to break a printlet with layers printed at a 0° axis. This was the case for both the 50% and 75% infill printlets (Figure 6c). The printlets printed using both printers, when tested after being placed vertically, collapsed on their structure without showing any breakage post removal and had their height reduced due to compression because of the applied force (Figure 6b). For both prints, breakages or crushing of the printlets was seen for 4 and 5 units for the continuous and batch processes, respectively. This can be attributed to the large void spaces and weak internal structural strength of the print at low infill densities.

### 3.6. Performance Comparison Using In Vitro Release Study

The HPMC AS used in the study here has a high number of acetyl and succinyl substitutions, which create a pH threshold for solubilization [26,35]. In the case of the LG grade, this threshold is the lowest as compared to its other grades MG and HG, i.e., pH > 5.5 [36,37]. As a result of this, a complete release was observed in this study where the release media used was phosphate buffer (pH = 6.8). The complete release for all batches was observed in the first few hours of the study. The infill density had a major impact on the drug release from the printed tablets, with a higher infill percentage having a slower release profile irrespective of their release mechanism. These findings were in line with the results of the study conducted by Thakkar et al. [38]. 

**Figure 7:**
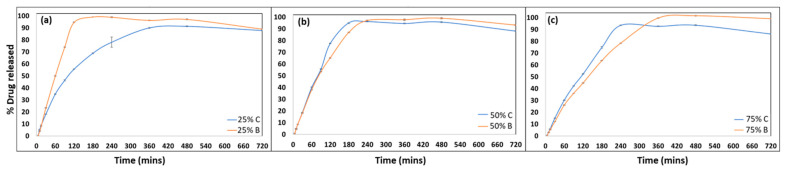
In vitro drug release performance comparison: (**a**) 25% infill printlets, (**b**) 50% infill printlets, and (**c**) 75% infill printlets, printed using continuous printing process (C) and batch printing process (B). The data are represented by mean ± standard deviation for n = 3 samples.

At a 25% infill percentage for the prints from both methods, the release rates are visibly different (Figure 7a), with the printlets from the batch process (25B) showing a faster release as compared to those printed using the continuous method (25C), which may be attributed to the layer orientation that changes the exposed surface area of the printlets to the neighboring release media. In the case of the continuous printing method with the printing axis and layer orientation at 45°, the bottom and the top surface of the printed tablets were completely covered and tightly packed, leaving no void opening for the media to penetrate in and interact with the matrix (Figure 7b,c). This packing remained the same irrespective of the infill density and was a characteristic of the layer orientation and printing axis. On the other hand, the printlets were printed in a batch fashion with a 0° layer orientation, and the internal pores of the prints were open and exposed to the surrounding media, which came in contact with the print as soon as they were introduced into the media. This could have been avoided if the top and bottom surfaces were covered with another surface layer. 

As stated earlier, a difference factor (*f*_1_) close to zero (≤15) indicates minimal differences between the curves and a similarity factor (*f*_2_) close to 100 (≥50) indicates closeness between the values of the test and reference samples [39,40]. Calculating these values to compare the release from 25C and 25M batches (Figure 7a), we obtained *f*_1_ = 20.86 and *f*_2_ = 36.95 (Table 2). This attests to the fact that these curves are different, and the release was impacted by the orientation at a 25% infill percentage. 

## 4. Discussion

Most of the studies published and currently ongoing in the field of FDM-based 3D printing are currently focused on diversifying the process for developing dosage forms and devices for pharmaceutical drug delivery applications [13]. From combining multiple drugs in the same printlet to innovating new strategies to better deliver a single therapeutic for the ongoing studies, multiple applications are being explored under different avenues [3,41]. However, most of these studies focus on developing a singular personalized medication for the patient to better cater to their needs and tailor the treatment for the diseased indications accordingly [1]. The approval of Spritam^®^ by Aprecia Pharmaceuticals and the recent patent licenses and FDA approvals for Triastek show that the application of 3D printing is not only limited to developing personalized medications but can also be extrapolated to target the R&D phase and clinical trial phase within a large patient populace [42,43]. 

The major limitation associated with FDM-based 3D printing is the time associated with the single printing process, which limits the viability of this process for scaling-up as compared to its counterparts [43,44,45,46]. With the current study, we established a continuous printing system and were able to bring down the print time to less than half of the time associated with the conventional FDM printing systems used in the pharmaceutical space. Both the printing time and lag time associated with the pre-and post-printing stages were eliminated using the setup in this study. While other studies have explored the use of exchangeable print beds to reduce the printing time and streamline the process, the exchange still requires manual intervention to remove the printed objects from the bed. The modified setup used in this paper eliminates the need for manual intervention while opening the capabilities to add in-line quality controls to the same system [47]. The adaptation to a continuous processing space while incorporating process control over the final formulation puts pharmaceutical 3D printing in an advantageous position to set itself up in line with the regulatory requirements and future changes [19]. The removal and collection of the printed tablets in this case were automated, as the printlets detached from the surface and fell off as they approached the end of the conveyor belt due to the curling at the round end of the belt at the scraper. This automated detachment reduces the chances for deformation after the application of force when removed manually, and thus overcomes that challenge. This makes this continuous process free from any manual intervention, which further helps comply with the regulatory guidelines and improve the healthcare space. Furthermore, in-line processing and quality control systems can be integrated into this processing system. This will help to monitor the quality of filaments and the final printed dosage forms as well [48,49]. The adaptation of Fourier transform near-infrared (FT-NIR) spectroscopy, a widely employed process analytical tool (PAT), will further improve quality control for the same system. The incorporation of a 2D laser triangulation scanner as shown by Faes et al. will help achieve both quantitative and qualitative information about the printed dosage forms. This will improve the robustness of the manufacturing system [50]. While understanding how additive manufacturing will affect the drug delivery field, setting up and modulating processes to best suit the needs of the pharmaceutical sector will help to avoid delays in real-world applications of the same system. 

The present study aimed to isolate a drug and polymeric matrix and focused on understanding the effect of layer orientation along with the two printing processes. We used a BCS class II drug to form an ASD using HPMC AS LG polymer, wherein we dispersed the drug at the molecular level in the polymeric matrix. Moreover, HPMC AS LG stabilized fenofibrate, a weakly acidic drug used in this study, in ASD through weak intermolecular interactions. This, however, may change as the drug load increases, which is associated with drug recrystallization at the surface as studied by Que et al. [51]. The capability of the polymer to maintain the supersaturation to increase the apparent drug solubility might go down as the drug loading increases [38,51]. During the HME optimization process, it was shown that at higher feed rates and higher screw speeds (rpm), the pressure built on the die increased, which rendered the extruded filaments unsuitable for printing. This unsuitability was attributed to the non-uniform diameter distribution and the non-flexible nature of the filaments due to the high-melt viscosity of the polymeric matrix. The optimization process was carried out at a feed rate of 3 g/min and a screw speed of 50 rpm. The extrusion state parameters, such as torque and die pressure, were recorded during the entire extrusion process and the filament sample was collected when the extrusion reached its steady state, where the die pressure (60 ± 10 psi at equilibration) and torque (4.80 ± 0.57 N·m at equilibration) were maintained at a steady rate. 

The continuous 3D printing setup printed the tablets at a 45° angle, which affected the tensile properties of the final print when we compared it to the conventional prints. However, when the infill percentage increased, the layers were more tightly packed in both prints. This reduced the void spaces available for the media to enter through and increased the release rate. In this case, the release worked by first forming a thin gel layer around the tablet surface, which was then followed by the complete solubilization of the matrix to achieve a complete drug release. In the case of HPMC AS LG, this gel layer formation does not play a significant role as compared to the MG and HG grades, which have a higher percentage of acetyl groups [26,38]. Thus, the release mechanism is thoroughly driven by the swelling, solubilization, and breakdown of the polymeric matrix to release the entirety of its content [38]. The differences between the curves minimize and the plots at 50% infill and 75% infill tend to be similar as can be seen from the *f*_1_ and *f*_2_ values in Table 2. The ASD formed was also able to maintain the increased apparent solubility of our drug over time, which can be attributed to the favorable miscibility of the drug-polymer as calculated using the Hansen parameter mentioned earlier, which in turn is supported by our solid-state characterizations. 

The release performance of the printed dosage forms was also affected at a lower infill but was not significantly different when evaluated at higher infill percentages. The release in the case of the HPMC AS LG was governed by the solubilization or hydration of the polymer and then dictated by the infill density and the printing orientation as established in this study [26]. This again may have been affected by the fact that the release profiles are governed by the polymeric matrix of interest, and understanding the effects of this printing parameter on different polymeric matrixes is something that needs to be explored to gain a better understanding [52,53]. 

The present study provides an in-depth understanding of the effect of layer orientation on the final dosage form, which is something that needs to be considered when moving to this setup. Optimizing the printing speed and temperature to match the setup to obtain a streamlined process without interruptions and clogging needs to be carried out while setting up the system. This study also highlights the importance of understanding the process parameters and their effects on the final dosage form before switching platform technologies. Further studies and advanced characterizations are necessary to understand the effect of other parameters that change when moving to a continuous printing platform.

## 5. Conclusions

This study demonstrated the first application of a continuous FDM-based 3D printing process in developing consistent batch-to-batch pharmaceutical dosage forms. The use of the dynamic *z*-axis ‘infinite’ printing space enables mass production/printing. The application of this novel continuous FDM-based 3D printing system is only limited by the feeding material, drug-loaded filaments in this case, which can be replenished without any manual intervention during the process. We were thus able to show for the first time the continuous FDM-based 3D printing of pharmaceuticals. The continuous printing process brought down the print time to less than 3 min per print for a conventional-sized tablet. The study also evaluated the print performances and reported that the differences between the printed dosage forms were minimized as the infill density increased. Furthermore, the system size and capacity can be modified to suit needs that could range from individualized patient-centered point-of-care machinery to a large-scale process for mass manufacturing. 

## Figures and Tables

**Table 1:** Dimensional measurements of printed dosage forms (n = 10) printed using continuous printing process (C) and batch printing process (M).

Process	Diameter (mm)	Height (mm)
Batch printing	7.843 ± 0.294	4.967 ± 0.199
Continuous printing	8.007 ± 0.055	5.004 ± 0.018

**Table 2:** Comparison between printlets’ release performance (n = 3) printed using continuous printing process (C) and batch printing process (M), using the similarity and difference factor method.

Reference	Test	Difference Factor (*f*_1_)	Similarity Factor (*f*_2_)
25B	25C	20.862	36.982
50B	50C	5.815	65.517
75B	75C	13.437	54.846

## Data Availability

Not applicable.
